# In silico modeling of anterior foregut endoderm differentiation towards lung epithelial progenitors

**DOI:** 10.1038/s41540-026-00650-1

**Published:** 2026-01-26

**Authors:** Amirmahdi Mostofinejad, David A. Romero, Dana Brinson, Thomas K. Waddell, Golnaz Karoubi, Cristina H. Amon

**Affiliations:** 1 Department of Mechanical and Industrial Engineering, University of Toronto, Toronto, ON Canada; 2 Institute of Biomedical Engineering, University of Toronto, Toronto, ON Canada; 3 Latner Thoracic Surgery Research Laboratories, Toronto General Hospital Research Institute, University Health Network, Toronto, ON Canada; 4 Institute of Medical Sciences, University of Toronto, Toronto, ON Canada; 5 Department of Laboratory Medicine and Pathobiology, University of Toronto, Toronto, ON Canada

**Keywords:** Developmental biology, Stem cells, Systems biology, Engineering, Mathematics and computing

## Abstract

Directed differentiation of human induced pluripotent stem cells (iPSCs) into anterior foregut endoderm (AFE) and lung progenitors (LPs) has wide-ranging implications for lung developmental biology, disease modeling, and regenerative medicine. We expand on a previously developed mathematical modeling framework and apply it to the directed differentiation of AFE into LPs. A model-based approach guides experimental design, followed by a multistage model inference process: maximum likelihood estimation based on in vitro data and identifiability analyses to eliminate unidentifiable candidates, thereby guiding model selection. To the authors’ knowledge, this is the first mathematical model of the population dynamics of directed differentiation of AFE into LPs. The model suggests that the overall dynamics are primarily driven by AFE proliferation and differentiation into LPs. In silico experiments predict that daily media change nearly doubles LP yields compared to cultures without media replenishment. Moreover, the model suggests that higher split ratios on day 10 enhance yield per input cell, a measure of differentiation efficiency, by 26%. This work provides a blueprint for refining iPSC-based lung lineage differentiation protocols by combining empirical data and mathematical modeling.

## Introduction

The differentiation of induced pluripotent stem cells (iPSCs) into lung epithelial progenitors (LPs) is critical for developmental biology studies and regenerative medicine applications. LPs give rise to alveolar^1^ and airway^2^ epithelium, both essential for lung function^3^. During directed differentiation, iPSCs are exposed to small molecules that mimic the signaling pathways guiding cell fate during fetal development. Definitive endoderm is first specified, followed by anterior foregut endoderm (AFE)^4^. Activation of Wnt and supplementation of BMP4 and retinoic acid in AFE yields LPs, which can be further differentiated into airway or alveolar lineages^5,6^.

Applications of directed iPSCs differentiation protocols are wide-ranging, especially in the fields of cell therapy and tissue engineering, where billions of cells are needed to attain clinically relevant grafts and treatments^7^. These protocols typically require the addition of a series of small molecules, growth factors, and reagents. Many of these protocols require several weeks or even months to generate the desired cell types^5,6^. Optimization of timing, cell density, and media formulation, among other factors, would be beneficial for most directed differentiation protocols, as it maximizes the desired cell types yields^8^. The design of differentiation protocols has historically been guided by developmental biology and relied predominantly on empirical studies, which can be costly, time-consuming, and suboptimal^9^.

Mathematical models can formulate experimentally testable hypotheses, guide the design of experiments, and be utilized in scaled-up clinical applications^10,11^. These models also have the potential to complement our understanding of complex biological phenomena, yielding an understanding of the dynamics that result in the presence of undesired non-lung endodermal lineages such as intestine, liver, or stomach when differentiating to distal LPs^12^. In silico modeling further complements in vitro experimentation by allowing researchers to test various culture conditions computationally, thereby reducing the number of experimental iterations^11^. Moreover, each iPSC line can respond differently to the same differentiation protocol, as mentioned by Jacob et al. NKX2-1^+^ LP yield can be between 30% and 90%, depending on the cell line^6^. This necessitates precise adjustments of culture parameters and timelines, which can be facilitated by mathematical models^13,14^. Such tailoring is especially critical for personalized medicine, where patient-specific iPSCs often demand custom approaches to achieve optimal cell populations^15^. Given these advantages, designing mathematical models to predict the differentiation and growth kinetics of AFE cells into LPs is essential to improve our capacity to optimize experimental conditions for generating lung tissues^15^.

In this paper, a previously reported model development approach is augmented to include biochemical effects alongside multicellular populations^16,17^. Due to the intrinsic differences between the physical units and measurement errors associated with cell density and substrate concentrations, independent error parameters are defined^18^. This model serves as a tool for further understanding the directed differentiation of iPSCs to LPs and enabling optimization of its protocols.

This is the first population dynamics model of directed differentiation of AFE to LPs, to the authors’ knowledge. Model inference starts with multiple biology-informed model proposals, considering two approaches to cell populations: one incorporating only the total population and one incorporating AFE and LP populations individually. We then perform model calibration and selection using in vitro observations from designed experiments and ensure model identifiability using mathematical tests. The inferred model is then validated by calculating the goodness of fit on the hold-out dataset. The model is then used to study the effects of different day 10 split ratios and the importance of split ratios and media refreshment protocols during culture.

## Results

**Fig. 1:**
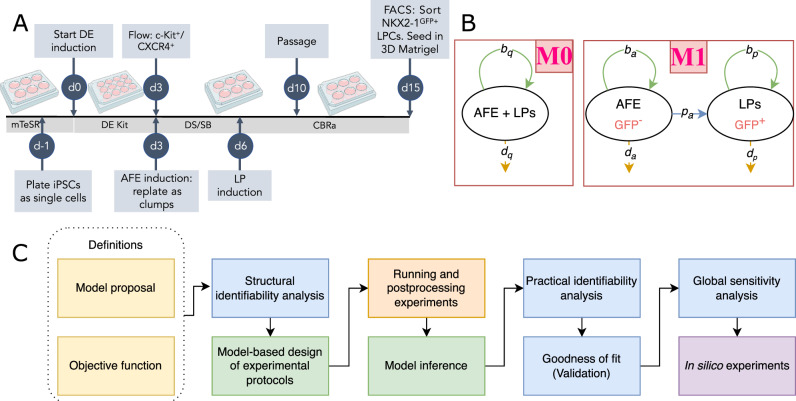
Experimental protocol and the lineage models. **A** Typical experimental protocol for directed iPSCs differentiation to lung progenitors. Also, mTeSR, DE Kit, FACS, DS, and SB represent mTeSR-1 iPSC maintenance medium, STEMdiff™ Definitive Endoderm Kit, fluorescence-activated cell sorting, dorsomorphin, and SB431542, respectively. We focus on model inference for days 10 to 15 of the directed differentiation process. Created in BioRender. Brinson, D. (2025)^82^. **B** Lineage models. AFE and LPs are anterior foregut endoderm and NKX2-1^+^ lung progenitors, respectively. Biomarker expressions are shown in red text. Green, blue, and orange lines correspond to proliferation, differentiation, and death rates, respectively. **C** Model development protocol. Yellow and blue boxes show the initial definitions and mathematical tests. Green boxes indicate the steps with model inference. Model-based design of experiments involves inference with synthetic data for experimental design, whereas model inference involves inference with in vitro data. This methodology is influenced by earlier work by the authors^16^.

This paper is organized around the experimental window in which AFE cells are induced toward NKX2-1^+^ LPs (Fig. 1A). Specifically, we focus on days 10–15 of the protocol, and the corresponding model development steps used to quantify this process, and use the model to predict the differentiation dynamics under modified culture conditions. Another paper by the authors performs a similar analysis for days 0–3 of this protocol^17^.

Two lineage formulations are considered in the model development stage (Fig. 1B): a one-population model that captures the total live cells (M0) and a two-population model that explicitly resolves the AFE and LP populations (M1), describing the differentiation of AFE to LP. Both models consider glucose and lactate as nutrients and waste products and evaluated under different growth and environmental effect hypotheses. Error models are also used to describe the difference between experimental observations and the mean (structural, shown in Fig. 1B) model.

Figure 1C depicts the workflow in this paper. We first define various candidate models and screen them using structural identifiability test. The next step is model-based design, followed by running experiments to obtain the necessary measurements. The collected data is used for model calibration and selection, and to validate the selected model. Parameter uniqueness is ensured, and global sensitivity analysis is used to understand the population system’s dynamics. The in silico model is then used to predict the effects of split ratio and media refreshment on LP yield and differentiation efficiency.

### Structural identifiability analysis

Structural identifiability is a property of a mathematical model indicating that, if we could measure the system perfectly (i.e., with no measurement error) and as often as needed, the model parameters would be uniquely determined^19–21^. Structural identifiability analysis was performed on all candidate models with the observables being the state variables in Eqs. (6) and (8). We showed that all but two of these models are globally structurally identifiable (StructuralIdentifiability.jl)^22–24^. The two unidentifiable structural models are M0 and M1, with exponential growth and without glucose or lactate effects. These two models are discarded, and the rest (22 remaining structural models) are used for parameter inference. Having restricted attention to structurally identifiable candidates, we then determined the measurement frequency required for reliable parameter estimation.

### Model-based design of experimental protocols

At this stage, we applied model-based design of experimental protocols (MBDEP) to determine the experimental details required for model inference. Since we assumed spatial homogeneity in the cell populations in the well plate, the population dynamics system simplifies to a temporal problem. In each experimental condition, the key design parameter is the measurement frequency (sampling period)^16^.

Based on our experimental capabilities, the sampling period could range from 0.5 to 4 days. Note that this design assumes four experimental conditions: two different split ratios and two settings, with and without media change. Here, it is assumed that the AFE differentiation can be well described by Eq. (8) with Gompertz growth and proportional noise of 30% (*b*_*n*_ = *b*_*c*_ = 0.3 in Eq. (13)). Next, we assume the model parameters are known from our prior understanding of the differentiation process; the model with these parameter values yields qualitatively similar dynamics to those observed in our previous experimental data (Supplementary Table 2).

**Fig. 2:**
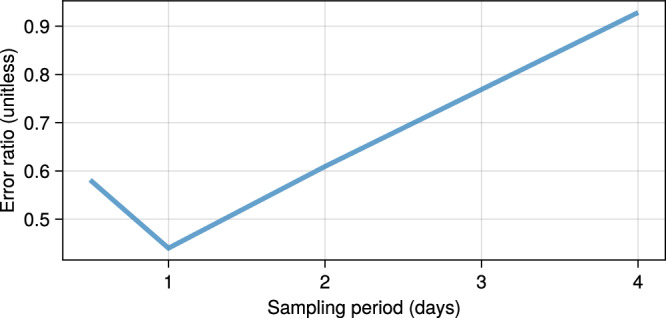
Parameter inference errors for different sampling periods. Sampling periods below 48 h are acceptable for this experiment.

Then, we used the assumed model and its parameters to generate synthetic data, perform parameter inference, and determine which sampling period yields the smallest distance (error) between the assumed and inferred parameters. The resulting error in parameter inference for the observables is plotted as a function of sampling frequency in Fig. 2. Estimating parameters for the set of model proposals considered here, taking individual live cell population measurements every 24 and 48 h, result in 44% and 61% error, respectively. For our experiments, we decided to sample concentrations and individual populations at 24 and 48 h, respectively, to balance experimental cost and model inference accuracy (Supplementary Table 1).

### Running and postprocessing experiments

As described in the experimental setup, the cells are passaged at two ratios of 1:2 and 1:5, resulting in different initial AFE cell populations (*N* = 4). This experiment, with daily media changes (MCH1), yielded total individual cell populations on alternate days and daily measurements of glucose and lactate concentrations. Additionally, a parallel experiment was conducted without any media change (MCH0), and similar measurements were recorded. Note that this results in 16 data points for the populations (2 in time, 2 for the AFE and LP populations, 2 for plating ratios, and 2 for media change) and 32 data points for concentrations (4 in time, 2 for the glucose and lactate, 2 for plating ratios, and 2 for media change), resulting in 48 total data points for each replicate.

Two-way statistical analysis of variance (ANOVA) test (using the Pingouin^25^ Python library) demonstrated statistically significant differences in terminal concentrations (day 15) between different initial populations and media change condition experiments. Similarly, the split ratio significantly affected some cell populations on day 15, suggesting that the experimental conditions affected the terminal (day 15) live LP cell populations. The ANOVA test confirms that the experimental data supports a connection between media refreshment and populations, corroborating environmental effect growth models.

### Model inference

We inferred the parameters for the candidate models using the calibration dataset (2:1:1 split for calibration, selection, and validation, respectively). The candidate models are M0 and M1 (named lineage models, stating the dynamics regarding distinguishable cell populations, refer to the Structural models section), with 11 growth models and three error models, resulting in 66 total models. Note that M0 and M1 have 6 and 9 parameters each, logistic and Gompertz growth models add one parameter, ${n}_{\max }$, and each biochemical effect adds one parameter, the corresponding *K*_*g*_ or *K*_*l*_, so models range between 6 to 12 structural parameters and error models add 2 to 4 extra parameters.

To guarantee thorough coverage, we used a maximin Latin hypercube^26^ to choose 100 starting points for the optimization. This space-filling sampling scheme selects initial parameter guesses that are as evenly distributed as possible across the allowed ranges, reducing the risk of missing good solutions. This resulted in 100 × 66 = 6600 optimization runs. A hypercube to draw the initial guesses for the optimization runs had the bounds [10^−4^, 10] for rates (unit being d^−1^), [0, 1] for differentiation ratio (unitless), and $[{n}_{\max }^{u}/10,{n}_{\max }^{u}]$ for ${n}_{\max }$. The upper bound of the latter, ${n}_{\max }^{u}$, is the maximum number of cells with a diameter of 15 μm that can occupy the entire area of the well plate bottom as a monolayer. Table 1 summarizes all the parameter search bounds.

**Table 1:** Parameter search space for our models

Definition	Parameter	Bounds	Unit
Average growth rate	*β*_*q*_	(0.0001, 10.0)	d^−1^
Average death rate	*δ*_*q*_	(0.0001, 10.0)	d^−1^
AFE proliferation rate	*β*_*a*_	(0.0001, 10.0)	d^−1^
AFE renewal ratio	p_*ap*_	(0.0, 1.0)	dimensionless
AFE death rate	*δ*_*a*_	(0.0001, 10.0)	d^−1^
LP proliferation rate	*β*_*p*_	(0.0001, 10.0)	d^−1^
LP death rate	*δ*_*p*_	(0.0001, 10.0)	d^−1^
Glucose reaction constant	*V*_*g*_	(0.0001, 1.0)	cell mmol L^−1^ mm^2^ d^−1^
Lactate reaction constant	*V*_*l*_	(0.0001, 1.0)	cell mmol L^−1^ mm^2^ d^−1^
Glucose proliferation MMK constant	*K*_*g*_	(0.01, 50.0)	mmol L^−1^
Lactate proliferation MMK constant	*K*_*l*_	(0.5, 200.0)	mmol L^−1^
Glucose reaction MMK constant	${\overline{c}}_{g}$	(0.0001, 50.0)	mmol L^−1^
Lactate reaction MMK constant	${\overline{c}}_{l}$	(0.0001, 50.0)	mmol L^−1^
Maximum density	${n}_{\max }$	(565.8, 5659.0)	cells mm^−2^
Density additive constant	*a*_*n*_	(0.1, 500.0)	cells mm^−2^
Density proportional constant	*b*_*n*_	(0.001, 2.0)	dimensionless
Concentration additive constant	*a*_*c*_	(0.1, 20.0)	mmol L^−1^
Concentration proportional constant	*b*_*c*_	(0.001, 2.0)	dimensionless

**Fig. 3:**
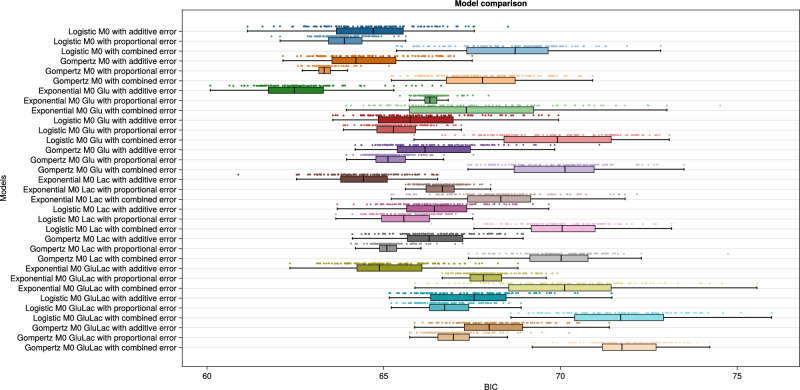
M0 model comparison. The rows show the 33 inferred models, with colors and shades representing structural and error models, respectively. Each row is derived from 100 parameter calibrations, each inferred from different initial guesses. The x-axis presents BIC values as discrete points, where a lower value indicates better model performance. These values are collectively summarized in the form of a boxplot.

**Fig. 4:**
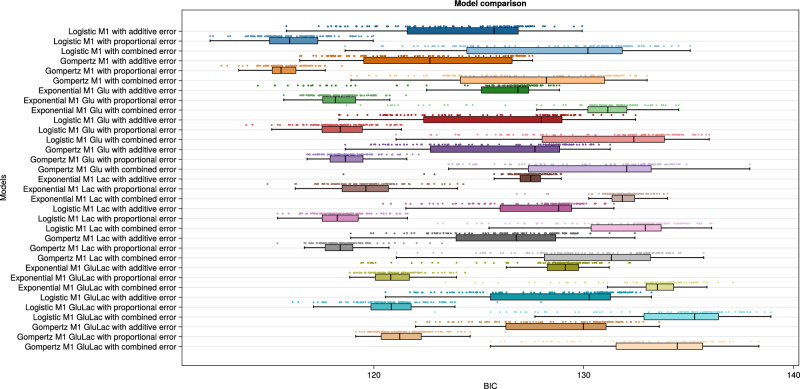
M1 model comparison. Note that the BIC value here is inconsistent with Fig. 3 since it includes two cell populations.

The two lineage models have different loss functions, resulting in incompatible BIC (Bayesian information criterion) definitions. This results in the two lineage models being compared separately, resulting in Figs. 3, 4 on the selection datasets. Model comparison on BIC values in Fig. 3 indicates the best-performing M0 model is exponential Glu M0 with additive error. It is important to note that the large spread observed in the boxplots for the BIC values reflects the inherent complexity and multimodality of parameter estimation in systems biology^27,28^, rather than the lack of convergence of the optimization algorithms.

Inferred parameter values and the corresponding confidence intervals are represented in Supplementary Table 3 for the four best-fitted inferred M1 models: Gompertz with proportional error, logistic with additive error, logistic with proportional error, and exponential Glu with additive error, in ascending order of BIC, according to Fig. 4. As can be seen from the table, two of the inferred values of ${n}_{\max }$ are unidentifiable, as observed by the upper bounds of the confidence interval not being found, indicating that it is either infinity or a very large value. The upper bound of ${n}_{\max }$ for logistic M1 with additive error is much greater than the physically defined upper bound shown in Table 1, making the parameter unidentifiable for this model. This observation shows that all models with ${n}_{\max }$ are practically unidentifiable. Population variations are minor in experiments, so the model might not see the existing effects of limitation by space. The existence of space-constrained growth could be observed with more experiments.

**Fig. 5:**
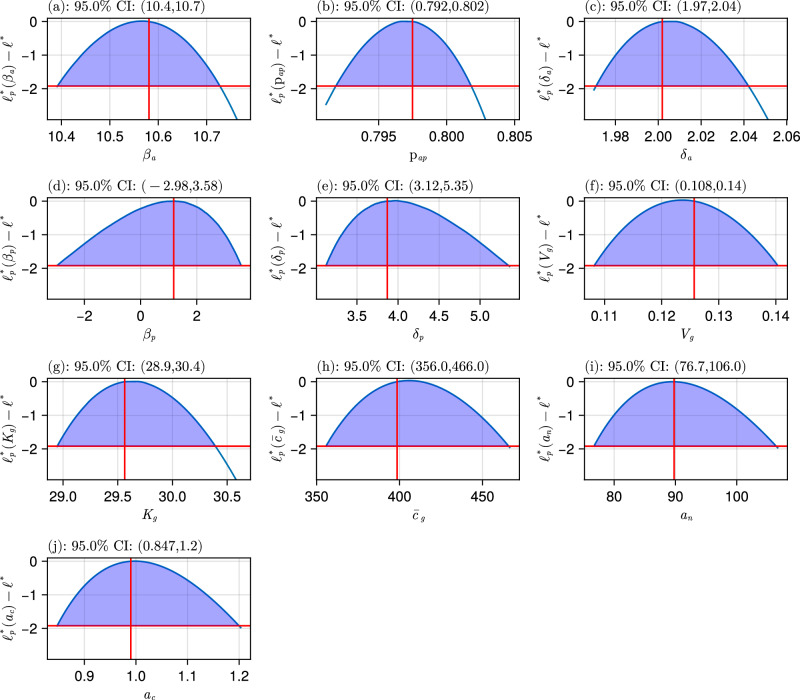
Likelihood profile for exponential M1 Glu with additive error model. Each x-axis corresponds to a parameter in the model, and the y-axis is the log-likelihood value. The intersection of the horizontal red line with the blue curve indicates each parameter confidence interval with 95% confidence, and the vertical red line is the inferred parameter value. Panels correspond to model parameters: (**a**) $\beta_a$, (**b**) $p_{ap}$, (**c**) $\delta_a$, (**d**) $\beta_p$, (**e**) $\delta_p$, (**f**) $V_g$, (**g**) $K_g$, (**h**) $\bar{c}_g$, (**i**) $a_n$, (**j**) $a_c$.

Observing the likelihood profiles for the models, Fig. 5, Supplementary Figs 3, 4, 5, it is evident that only exponential Glu M1 model has a concave downward profile, needed for a well-defined model in the proximity of the inferred parameters. The differentiation ratio, ${p}_{ap}$, is unidentifiable for logistic M1 with proportional error and Gompertz M1 with proportional error, and *β*_*a*_ is unidentifiable in the logistic M1 with an additive error model. This analysis yields exponential Glu M1 with additive error as the chosen M1 model, as it is the only practically identifiable model for all the parameters in the top four M1 models.

The BIC values in Fig. 4 can be compared with the loss values in Supplementary Fig 1. The loss values are similar to BIC values without the effect of the number of parameters; zeroing the first term in Eq. (18). This analysis shows that the negative effect of the number of parameters on the error measure, BIC, directs model selection towards model parsimony.

**Fig. 6:**
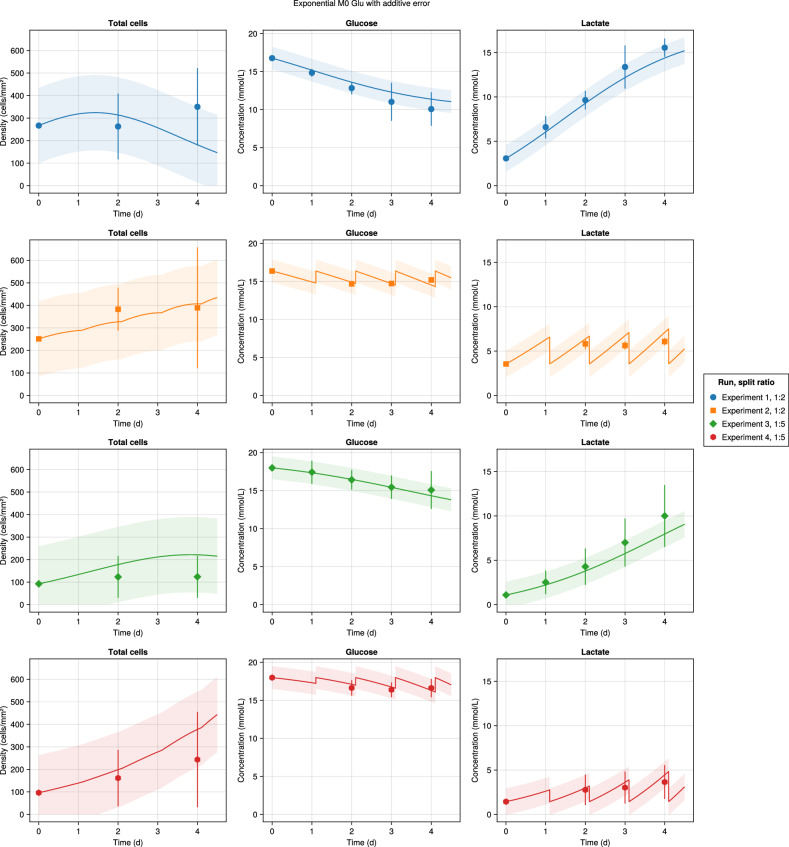
Experimental observations versus the model predictions for the exponential Glu M0 with the additive error model.

**Fig. 7:**
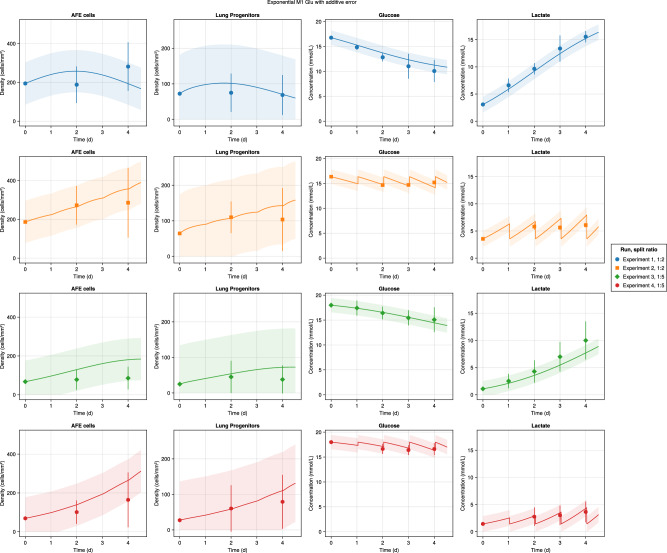
Experimental observations versus the model predictions for the exponential Glu M1 with an additive error model.

In silico model predictions versus the experimental observations in all experiments for the inferred M0 and M1 models are shown in Figs. 6, 7, respectively, with different colors representing the different experimental conditions; experiments one and three correspond to MCH0 culture, and experiments two and four to MCH1 culture. Markers show the mean values of the experimental data, and the error bars indicate the standard deviations (*N* = 4). The curves represent the inferred model expected values, while the bands show the inferred model standard deviations. Looking at the mean experimental measurements for M0, 87.5% of population measurements are inside the error model prediction, and the number is 96.4% for concentration measurements, displaying a good match between observations and the model predictions at most time points. A similar trend is seen with M1, with 100% and 92.8% coverage of the population and concentration measurements, respectively. The highest deviation between the model and prediction appears on day 4 of the total and AFE populations, meaning that the model overestimates the effect of glucose deficiency on growth rate reduction in higher populations and underestimates it in lower populations.

### Practical identifiability analysis

Practical identifiability extends structural identifiability analysis to real, limited experimental data subject to measurement error. In practical terms, it describes how sensitive the model fit is to changes in parameter values: an identifiable model has a clear “best fit," meaning that even small parameter changes noticeably worsen the fit^29^. The parameter confidence intervals are calculated by confining each parameter and minimizing the loss function^17^. Studying the width of the confidence intervals develops insights into the quality of model inference^30^. We performed profile likelihood-based practical identifiability analysis using the ProfileLikelihood.jl^31^ package.

Table 2 shows the inferred parameters and the resulting confidence intervals for all estimated model parameters for inferred M0 and M1 models. A few observations from the inferred error parameters can be driven from the inferred values of error model parameters, *a*_*n*_ and *a*_*c*_. Both models have the same concentration state, glucose, meaning that similar values for the additive standard deviations are expected, corroborated by the two inferred *a*_*c*_ values not being significantly different, as shown in Table 2. On the contrary, the population states are different between the models, total population for M0, and AFE and LP populations for M1, with total population defined as the sum of the two individual populations, *n*_*q*_ = *n*_*a*_ + *n*_*p*_. So, in the case of dependence of *n*_*a*_ and *n*_*p*_ with the correlation coefficient ${\rho }_{{n}_{a}{n}_{p}}$, the standard deviation of *n*_*q*_ is defined as,

1 $$\begin{array}{rcl}{a}_{{n}_{{\rm{M}}0}} & = & {\sigma }_{{n}_{q}}=\sqrt{{\sigma }_{{n}_{q}}^{2}}=\sqrt{{\sigma }_{{n}_{a}}^{2}+{\sigma }_{{n}_{p}}^{2}+2{\rho }_{{n}_{a}{n}_{p}}{\sigma }_{{n}_{a}}{\sigma }_{{n}_{p}}}\\ & = & \sqrt{2{a}_{{n}_{{\mathrm{M}}1}}^{2}(1+{\rho }_{{n}_{a}{n}_{p}})}=\sqrt{2(1+{\rho }_{{n}_{a}{n}_{p}})}{a}_{{n}_{{\mathrm{M}}1}}\end{array}$$

where *σ* is the standard deviation, and ${a}_{{n}_{M0}}$ and ${a}_{{n}_{M1}}$ are the error parameters of models M0 and M1, respectively. This means that looking at the two inferred *a*_*n*_ values from Table 2, the value for the correlation coefficient is 32%, which is comparable with the correlation coefficient calculated from raw data, 45%. These observations on the error model parameters indicate their consistency and support the correctness of the model inference process.

**Table 2:** Value and confidence intervals (lower bound, higher bound) for inferred models

Parameter	M1		M0		Unit
	Value	CI	Value	CI	
*β*_*q*_			8.209	(5.090, 27.74)	d^−1^
*δ*_*q*_			1.710	(1.238, 4.034)	d^−1^
*β*_*a*_	10.58	(10.39, 10.73)			d^−1^
p_*ap*_	0.7975	(0.7919, 0.8018)			dimensionless
*δ*_*a*_	2.002	(1.971, 2.042)			d^−1^
*β*_*p*_	1.178	(-2.976, 3.581)			d^−1^
*δ*_*p*_	3.869	(3.121, 5.350)			d^−1^
*V*_*g*_	0.1257	(0.1082, 0.1404)	0.2059	(0.01002, 1.823)	cell mmol L^−1^ mm^2^ d^−1^
*K*_*g*_	29.56	(28.95, 30.39)	52.70	(38.76, 488.2)	mmol L^−1^
${\overline{c}}_{g}$	398.5	(355.6, 465.8)	662.4	(17.94, 5000)	mmol L^−1^
*a*_*n*_	89.77	(76.70, 106.4)	145.8	(115.5, 198.3)	cell mm^−2^
*a*_*c*_	0.9900	(0.8472, 1.200)	0.9732	(0.8045, 1.185)	mmol L^−1^

Both models are Exponential Glu with additive error models.

Figure 5 shows the likelihood profiles for the inferred M1 model. Note that the red vertical and horizontal lines correspond to the inferred parameters and the 95% confidence threshold, respectively. The intersections of the curve and the horizontal line show the lower and upper bounds. The figure indicates finite, relatively narrow confidence intervals, consistent with practical identifiability of the model based on our experimental data^32^. On the contrary, the likelihood profiles for M0 are depicted in Supplementary Fig 2, showing a few unidentifiable parameters.

In summary, this analysis shows that the M1 model, while adding three parameters and needing more measurements provided by flow cytometry, resulted in an identifiable and more detailed model with two cell populations. The rest of the paper focuses mainly on exponential M1 Glu with additive error as the chosen model. Finally, it is noted that the confidence interval for *β*_*p*_ in the model includes zero, meaning that the LP proliferation effect may not be statistically significant based on the experimental data and that the system’s dynamics might be entirely driven by the proliferation and differentiation of the AFE population.

### Goodness of fit

The root-mean-square prediction error (RMSE) of the inferred model was calculated using the validation (hold-out) dataset. Results show that the inferred model has an RMSE of 102.3 cells mm^−2^ for cell densities. For context, raw data and inferred standard deviations are 104.46 and 89.77 cells mm^−2^, respectively. Given that the model RMSE is comparable to the experimental variance in the data, we consider the model sufficiently accurate for its applications in supporting AFE differentiation to LPs.

Furthermore, to estimate the predictive accuracy of the model over unobserved experiments, we performed leave-one-out cross-validation, holding out the entire time series (all time points) from all replicates of one experimental condition. The inferred model, exponential Glu M1 with additive error, is recalibrated on 12 of the time series datasets (related to 3 of the experiments), and the held-out experiment time series is used to assess the population prediction error. Normalized RMSE is calculated using Eq. (22) to be equal to 18.2 ± 8.8% (average and standard deviation), which is below the standard deviation of the experimental data (89.2%) and the 30% threshold that has been used in previous works^33^.

Similarly, RMSE, mean absolute error (MAE) and mean error (ME) across the folds are calculated as 87.5 ± 42.0 cells mm^−2^, 61.6 ± 24.4 cells mm^−2^, and 5.9 ± 26.3 cells mm^−2^, respectively. MAE provides the typical magnitude of the prediction error in the same units as the measurements, while ME quantifies directional bias; values near zero indicate no systematic over- or under-prediction. Here, the MAE indicates that predictions are typically within 62 cells mm^−2^ of the observations, and the near-zero average ME suggests that the model is not consistently biased in one direction across conditions. The higher RMSE relative to MAE suggests that, while typical errors are moderate, some held-out conditions or time points exhibit larger deviations^34^. This analysis reflects prediction accuracy for new experimental conditions in the split ratio interval [1:5, 1:2], not just interpolation between time points.

### Global sensitivity analysis

We conducted global sensitivity analysis of the exponential M1 Glu model. This analysis ranks model parameters by their impact on predictions, helping to identify model dynamics. In particular, we computed the sensitivity of the AFE and LP populations, as well as glucose concentration, to the structural model parameters. First-order Sobol indices rate the significance of parameters, while total-order Sobol indices additionally consider parameter interactions.

**Fig. 8:**
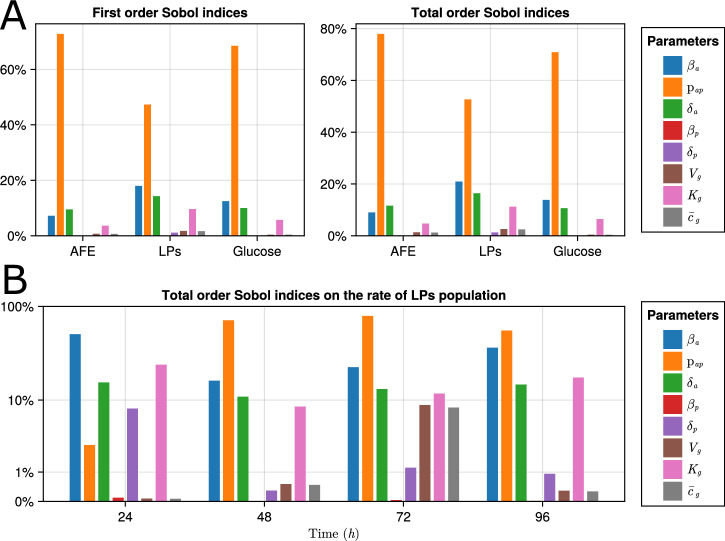
Global sensitivity analysis on exponential Glu M1 model. **A** GSA on day 4 values of observables. The x-axis shows the observables. **B** Time evolution of the total order Sobol indices of the rate of LP population to model parameters.

In order to achieve this, we employ 40,000 samples from the bounds [0.909, 1.10]θ^*^ for all the parameters with Sobol’s method^35^. Figure 8A shows the resulting Sobol indices for the split ratio of 1:2 with MCH0 culture at time *t* = 96 h, equivalent to experiment 1 in Fig. 7. The model predicts that parameter interactions are less significant than their first-order effects, as evidenced by the qualitative consistency between the first-order and total-order Sobol indices. Also, it predicts that proliferation and death rates of LPs have insignificant effects on the AFE population, as can be inferred from Eq. (8) by examining the effect of the LP population on glucose consumption. The negligible contribution of the LP proliferation rate, *β*_*p*_, to any of the states is consistent with confidence intervals from practical identifiability analysis. On the contrary, the model’s population growth is driven primarily by the differentiation ratio, ${p}_{ap}$, AFE proliferation, *β*_*a*_, and the death rate, *δ*_*a*_. The sensitivity analysis indicates that, at this stage of the differentiation protocol, population dynamics are predominantly driven by AFE cellular processes.

Figure 8B shows the time evolution of total-order Sobol sensitivity indices throughout the experiment for the LP population growth rate. The figure indicates that throughout the experiment, the LP population rate is driven by the AFE cellular process and glucose through the parameter *K*_*g*_. It is worth noting that glucose metabolism parameters, *V*_*g*_ and ${\overline{c}}_{g}$, do not directly affect the population, which accounts for their lower GSA values. The model predicts that the initial glucose concentration and media replacement rate affect differentiation; further explored in the following section.

### Applications

The primary motivation for developing mathematical models is their ability to run multiple in silico experiments quickly, enabling exploration of various protocols and cell growth conditions. These in silico experiments focus on the effect of day 10 split ratios on the growth dynamics by studying the defined response variables. Further, we study the effect of media change protocols by considering two conditions, no media change (MCH0) and daily media change (MCH1), to quantify the extent to which media change has enhanced the protocols.

As mentioned (Fig. 1A), the experimental procedure includes passaging the cells at day 10 of the protocol with a given split ratio and taking measurements of AFE and LP populations on days 11, 13, and 15. The cells need to be seeded between days 10 and 11, and as observed in our experiments, the live cell population drops significantly, showing a completely different set of dynamics between day 10 and day 11, compared with the dynamics between day 11 and day 15. The inferred model predicts the dynamics between days 11 and 15, with day 11 measurements as its initial conditions. The split ratio at day 10 can be directly controlled, while the subsequent day 11 populations are not directly controlled, they result from the split ratio and the growth environment.

To bridge this gap between the initial conditions of the model (not directly controllable) and the day 10 split ratios (directly controllable during plating), we introduced two linear functions to map the day 10 split ratios to day 11 populations, where SR is the split ratio (dimensionless). The inferred mappings between the split ratio, SR, and model initial conditions are defined as,

2 $$\begin{array}{rcl}{n}_{{a}_{0}} & = & {\kappa }_{1}{\mathrm{SR}}+{\kappa }_{{0}_{1}},\\ {n}_{{p}_{0}} & = & {\kappa }_{2}{\mathrm{SR}}+{\kappa }_{{0}_{2}}.\end{array}$$

Here, *κ*_1_, *κ*_2_, ${\kappa }_{{0}_{1}}$, ${\kappa }_{{0}_{2}}$ are estimated through robust linear regression (GLM.jl^36^) to be 407.845, 140.941, −13.2916, and −2.04044 cells mm^−2^, respectively. The coefficients in the split ratio interval [1:5, 1:2] show that on day 11 of the protocol, approximately 25% of the cell population is differentiated to LPs. The top plot in Supplementary Fig 6 depicts the initial conditions used in the in silico models, and the line shows the one-dimensional space explored to observe the effect of the split ratio. The bottom plots show the two mappings between the day 10 split ratios and each of the initial conditions.

**Fig. 9:**
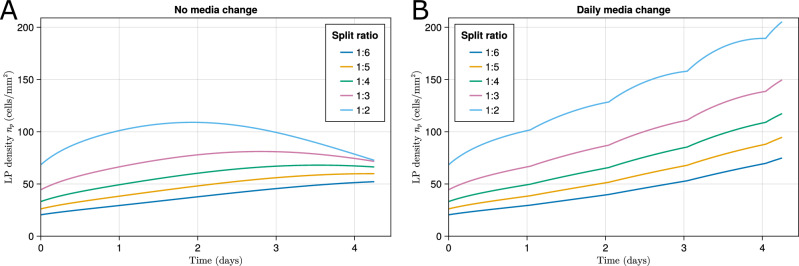
Time evolution of LP density in different split ratios and media change conditions. **A** No media change (MCH0). **B** Daily media change (MCH1).

The previous step generated initial conditions corresponding to target split ratios, producing the time evolution of LP densities shown in Fig. 9 for MCH0 and MCH1 cultures. The figure illustrates the model prediction that the MCH0 culture causes the LP populations to peak and then decline before four days of culture. It is also predicted that the fluctuations become more pronounced at higher AFE densities. This might be caused by the linear increase in glucose consumption with cell density, so glucose would be depleted faster at higher densities, leading to lower proliferation and differentiation rates and a swifter decline in cell populations. Production of lactate and consumption or degradation of other substrates, such as recombinant human Bone Morphogenetic Protein 4 (BMP4), retinoic acid (RA), and CHIR99021, have a similar direct relationship with respect to the cell population and might contribute to this behavior^6,37,38^. Supplementary Fig 7 shows the model prediction of the long-term behavior of the cell population, assuming the inferred dynamics hold. It is predicted that although exponential growth would result in no limit to the population, because the growth rate is also dependent on the glucose concentration, MCH1 culture would result in a maximum LP density of around 400 cells mm^−2^.

Quantification of the effect of the experimental conditions is done by defining response variables as,

3 $$\begin{array}{rcl}{n}_{{p}_{4}} & = & {n}_{p}(t=4),\\ {\rm{Y}}{\rm{i}}{\rm{e}}{\rm{l}}{\rm{d}}\,{\rm{p}}{\rm{e}}{\rm{r}}\,{\rm{i}}{\rm{n}}{\rm{p}}{\rm{u}}{\rm{t}}\,{\rm{c}}{\rm{e}}{\rm{l}}{\rm{l}} & = & {n}_{p}/{n}_{{q}_{0}},\\ {\mathrm{LP}}\,{\rm{r}}{\rm{a}}{\rm{t}}{\rm{i}}{\rm{o}} & = & {n}_{p}/{n}_{q}.\end{array}$$

Here, ${n}_{{p}_{4}}$ is the LP density at day 4 of the model (equivalent to day 15 of the protocol), yield per input cell is the ratio of LP density to initial total density, and LP ratio is LP density to total density.

**Fig. 10:**
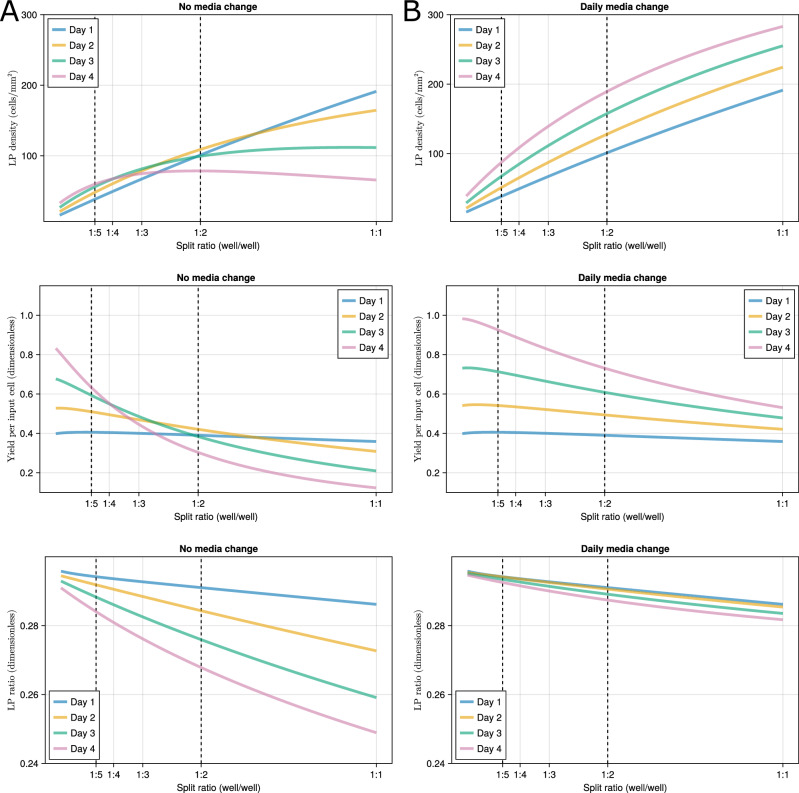
The effect of different day 10 split ratios. Each row shows the effect of the split ratio on one of the response variables. **A** No media change (MCH0). **B** Daily media changes (MCH1).

Traversing the day 11 initial condition space shown by the orange line on Supplementary Fig 6 and running simulations to calculate the response variables defined by Eq. (3) results in a larger scale comparison between the split ratios shown by Fig. 10. The vertical dashed lines denote split ratios of 1:2 and 1:5, the two ratios used in experiments, meaning predictions in this interval are interpolations. Figure 10A, B relate to the MCH0 and MCH1 cultures, respectively, and their rows stand for different response variables.

### Effect of media change protocol

To investigate the effect of media changes on cell population dynamics, we compared two experimental conditions, MCH0 and MCH1, as defined previously. Note that both conditions yield similar results on day 1, as the first media change is made immediately after the day 1 measurements (Fig. 10A, B). By day 4, however, as predicted by the model, MCH1 significantly enhances all three response variables, LP density, yield per input cell, and LP ratio, especially at lower split ratios where nutrient consumption is greater.

For example, in the [1:5, 1:2] split ratio interval, daily media refreshment is predicted to nearly double the day 4 LP density and yield per input cell (both increased by 94%), while moderately improving the LP ratio by 5.3%. The model suggests that more frequent nutrient replenishment supports larger overall populations without substantially altering their terminal proportion of cell types. A similar pattern emerges in the experimental data presented in Fig. 7, where MCH1 culture improves day 4 LP density and yield per input cell by 80% and increases the LP ratio by 21%. Considering the error parameter *a*_*n*_ (Table 2), these observed improvements closely align with the in silico results and are consistent with model predictions that MCH1 culture increases cell growth without altering the proportion of cell types.

### Effect of split ratios

In silico, we investigated the effect of split ratios on cell population dynamics of the MCH1 culture. The uppermost panel in Fig. 10B shows the model-predicted LP densities as a function of split ratios, indicating that lower split ratios increase LP density. In contrast, all the daily density plots are concave downward over the split ratio, predicting that the yield per input cell is decreased at lower split ratios, as also depicted by the middle row in Fig. 10B. In silico simulations suggest that the system’s efficiency decreases as the split ratio decreases, and the yield per input cell drops from 0.925 to 0.733 in the [1:5, 1:2] split ratio interval. As the lower row in Fig. 10B illustrates, the change is predicted to be mainly around the total population, and the proportion of the cell types does not change significantly. In summary, the model predicts that higher split ratios yield up to 26% higher efficiency on day 15 of the differentiation protocol. This is rather conservative compared to the experimental observations from Fig. 7, where a split ratio of 1:5 would nearly double (99% increase) the yield per input cell compared to the split ratio of 1:2. This is evidence that the model underestimates the effect of growth deceleration in higher populations caused by the selection of exponential growth over logistic and Gompertz growth.

## Discussion

This paper illustrates a mathematical model for the differentiation of AFE to LPs. The candidate models were structured using two lineage models (one- and two-state models), three growth models (exponential, logistic, and Gompertz), and the presence or absence of the MMK effect of glucose and lactate on growth, resulting in 24 structural models. This was complemented with three candidate standard deviation models individually defined for the cell densities and the substrate concentrations, resulting in 72 total models. The two individual error models are defined because the measurement methods are different, and human error plays a more significant role in cell density measurements. Six models were discarded because of the structural unidentifiability, and model calibration and selection were performed on the rest of the models using calibration and selection datasets. The best practically identifiable model was selected by analyzing the likelihood profiles of models with the best BIC scores. The inferred model was validated with the RMSE of 102.3 cells mm^−2^ compared with inferred standard deviations of 89.77 cells mm^−2^, indicating a sufficiently accurate model. All these steps showed the extensibility of the previously developed framework for equation definition and model inference^16,17^.

The sampling period for the experimental protocol is supported by MBDEP^16^. The in vitro experiments are conducted with and without growth media refreshment to ensure the cells are subject to resource-deprived conditions and the potential effect of the biochemical environment can manifest. Practical identifiability analysis of the inferred models showed that the individual populations obtained from flow cytometry not only assisted with constructing a more detailed model but also helped create an identifiable model with unique parameter values. A practical identifiability analysis step could be added to MBDEP to ensure model identifiability given the perceived measurement error and the design sampling period prior to running experiments.

Mathematical tests, such as practical identifiability and global sensitivity analyses, provided better insight into the experimental protocol. Both analyses predicted that cellular differentiation is more important than other dynamics, confirming that the AFE population is the main initial population affecting the terminal LP density. The former did this by not refuting the LP proliferation rate, *β*_*p*_, being zero, and the latter by showing a small sensitivity index for this parameter.

Directed differentiation results are sensitive to cell density, necessitating optimization of seeding density for different cell lines^39^. Cell density has been shown in several differentiation systems to influence pluripotency and cell fate. This occurs via paracrine signaling, cell shape, and metabolic activity^40–42^. Specifically, during the derivation of AFE into LPs, it is recommended to passage the cells between days 8 and 10 to avoid over-confluence^6^. The AFE differentiation model is applied to conduct in silico experiments to predict the effects of split ratios. The model suggested no significant effect of split ratios on the LP ratios. This observation is corroborated by Ptasinski et al.^43^, who show that split ratios between 1:6 and 1:3 do not significantly affect the day 15 LP ratio.

In silico experiments further suggested that decreasing the split ratio lowers the yield per input cell, a measure of the system’s efficiency, while it increases the LP density. A similar pattern has been observed in the directed differentiation of iPSCs to cardiomyocytes^44^, where a higher split ratio resulted in higher yields^44^. The yield per input cell might be an essential variable for optimization when working with scarce cells. However, since no candidate model accounted for the Allee effect^45^, which stipulates a minimum cell population needed for survival, extrapolating the results to higher split ratios is less reliable, meaning the model cannot suggest split ratios above 1:5.

Another in silico experiment was performed to investigate the effect of daily media changes on growth dynamics. Daily media changes prevent nutrient depletion and metabolite buildup^46^. Furthermore, the stability of small molecules and growth factors used in directed differentiation protocols is a concern because BMP4 and retinoic acid have limited half-lives under normal cell culture conditions, especially in serum-free media^47^. Because only 8% of added all-trans retinoic acid remains after 24 h of incubation with cells^48^, daily media changes help replenish this critical factor. The model predicts that, on average, daily media replacement nearly doubles LP density on day 15 relative to no media change, without significantly affecting population ratios. One possible explanation is that daily media changes likely improves differentiation efficiency by continually replenishing the small molecules required for directed differentiation. This analysis is consistent with experimental data, illustrating how the model can be used to study the effect of the biochemical environment on culture dynamics. Using the in silico model, the media change frequency and ratio (the fraction of media changed) can be explored, optimized, and prioritized for experimental testing.

As mentioned in the Model inference section, none of the inferred models with maximum density, ${n}_{\max }$, were identifiable. This might be because the populations were too small for this effect to appear. Future experiments could focus on split ratios below 1:2, the lower bound used in this paper. Also, the existence of a minimum population for the AFE to successfully differentiate into LPs, the Allee effect, can be explored^49^, needing experiments with split ratios higher than 1:5. Note that the model developed in this paper incorporated data from experiments with day 10 passaging. Future model inferences can include data from experiments without day 10 passaging to quantify the effect of passaging and further generalize the model. These would enable the inferred model to cover a broader experimental range and facilitate the search for a global optimum in split ratios.

The inferred model can be improved to become more descriptive by including a ventral anterior foregut endoderm (vAFE) transition state. Expression of NKX2-1, PAX1, and NKX2-5 can be used to indicate the population of vAFE^4^. Then, mathematical models similar to the M2 lineage model from the authors^17^ can be calibrated, and a similar model selection protocol can be utilized.

LPs are developmentally immature primordial cells, the first cells expressing biomarkers specific to the lungs^1^. There is a wealth of research on establishing protocols for producing iPSC-derived LPs^1,5,50,51^. It is shown that WNT (activated using CHIR99021), RA, and BMP4 are critical to lung specification^6^. Due to wide adoption by the regenerative medicine community and the utilization of CHIR, BMP4, and RA in many protocols for later-stage cells, e.g., type I and II alveolar epithelial^6^, airway organoids^2^, and purified basal cells^52^, this paper focused on differentiation to LPs using these small molecules. Future studies could incorporate cell signaling pathways by considering more states and observables representing biomarkers, inhibitors, catalysts, and proteins, thereby increasing the model’s explainability and its value for protocol optimization. Such model inference would require additional measurements from the newly defined observables to provide an identifiable model. This is done by conducting experiments with varying nutrient and growth factor levels and media replacement periods^53,54^.

In summary, this study highlights the potential utility of integrating in silico modeling to optimize AFE differentiation protocols. The model offers a refined approach for enhancing the production of LPs from AFE cells by quantifying the impact of key experimental parameters such as media refreshment and split ratios. This procedure is intended to support reproducibility and efficiency in generating clinically relevant cell populations.

## Methods

### Experimental setup

iPSCs were maintained as colonies on hESC-Qualified Matrigel (Corning, cat. no. 354277)-coated 6-well plates. Prior to differentiation, cells were first passaged as single cells onto Corning® Matrigel®-coated 12-well plates and cultured in the iPSC maintenance medium (mTeSR-1, StemCell Technologies, cat. no. 85850) for 24 hours with Y-27632 (10 μM, StemCell Technologies, cat. no. 72304). Then, the cells were cultured with STEMdiff™ Definitive Endoderm Kit (StemCell Technologies, cat. no. 05110) for 72 h.

On day 3, cells were dissociated as clumps using Gentle Cell Dissociation Regent (GCDR; StemCell Technologies, cat. no. 07174) and passaged onto Corning® Matrigel®-coated 6-well plates and cultured in DS/SB (cSFDM with 2 × 10^−6^ mol L^−1^ dorsomorphin (Tocris, cat. no. 3093) and 10 × 10^−6^ mol L^−1^ SB431542 (Tocris, cat. no. 1614)) with Y-27632 (10 *μ*M, StemCell Technologies, cat. no. 72304) medium for 24 h. Complete serum-free differentiation media (cSFDM) consisted of Iscove’s Modified Dulbecco’s Medium (IMDM; Gibco, cat. no. 12440053) and Ham’s F-12 (Gibco, cat. no. 11765054) supplemented with 0.5x B-27 (Invitrogen, cat. no. 17504001), 0.5x N-2 (Invitrogen, cat. no. 17502-048), 50 μg mL^−1^ ascorbic acid (Sigma-Aldrich, cat. no. A4544), 500 μg mL^−1^ monothioglycerol (Sigma-Aldrich, cat. no. M6145), 0.056% bovine albumin fraction V (Thermo Fisher, cat. no. 15260037), 1x Glutamax (Thermo Fisher, cat. no. 35050-061), and 50 μg mL^−1^ Primocin (Invivogen, cat. no. ant-pm-2). Subsequently, they were incubated for another 48 h in DS/SB medium without Y-27632 at 37 °C.

On day 6, the culture medium was switched to CBRa (cSFDM with 3 × 10^−6^ mol L^−1^ CHIR99021 (Tocris, cat. no. 4423), 10 ng mL^−1^ recombinant human bone morphogenic protein (BMP4; R&D Systems, cat. no. 314-BP-050), and 50 × 10^−9^ mol L^−1^ retinoic acid (RA; Sigma-Aldrich, cat. no. R2625)) medium. When the cells were confluent (typically at day 10), they were passaged at densities 1:2 and 1:5 into fresh Matrigel-coated 6-well plates containing CBRa medium and were incubated until day 15. Two culture conditions, one without and one with daily media refreshment, were run to observe the effect of the biochemical environment on the induction of lung progenitors (Fig. 1A).

Each day, media samples of 160 μL were taken from all wells and the glucose and lactate concentrations were measured using RAPIDPoint 500 Blood Gas Systems (Siemens Healthcare Limited, Canada). Then, one well per condition was harvested daily to measure total live, AFE, and LP populations. The measurements for each model, along with the time points at which they were collected, are shown in Supplementary Table 1. Note that the data collection frequency is determined by the model-based design of experimental protocols.

The wells were first rinsed with PBS (−/−). Cells were then treated with 0.05% Trypsin-EDTA (Wisent, cat. no. 325-542-CL) and incubated at 37 °C for 3 min. The empty plate was washed with Dulbecco’s Modified Eagle Medium (DMEM; Wisent, cat. no. 319-005-CL) supplemented with 10% Fetal bovine serum (FBS; Thermo Fisher, cat. no. 12483020) and 1% penicillin/streptomycin (Wisent, cat. no. 450-201-EL), which was then combined with the trypsinized cells. The cell mixture was centrifuged at 300 g for 5 min. The cell pellet was resuspended in DMEM with 10% FBS and 1% penicillin/streptomycin. A 20 μL aliquot of the resuspended cells was used for cell counting with a hemocytometer. Trypan blue (Gibco, cat. no. 15250061) was used to identify and count dead cells.

### Model proposal

The mathematical model incorporates two main components, the structural, **g**, and error, ***ϵ***, models. The two models are used to define the experimental observations, **z**, as,

4 $${\bf{z}}({\bf{Y}},{\mathbf{\Theta }},{\boldsymbol{\xi }},{\bf{u}})={\bf{g}}({\bf{Y}},{\mathbf{\Theta }},{\bf{u}})+{\boldsymbol{\epsilon }}({\bf{Y}},{\mathbf{\Theta }},{\boldsymbol{\xi }},{\bf{u}}){\boldsymbol{\eta }},$$

where ***η*** is the vector of normalized residuals, which are assumed to be independent random variables drawn from a Gaussian distribution with zero mean and unit standard deviation^18^. Also, **Θ,** ***ξ***, **Y,** **u**, are vectors containing structural parameters, error parameters, state variables, and external stimuli, respectively. Below, structural and error models are further discussed.

### Structural models

The structural model is composed of lineage and growth models. Lineage models focus on the cellular states and processes, while the growth models focus on the growth rates affected by different stimuli. Here, lineage models are discussed, followed by growth models.

As seen in Fig. 1B, we investigate two potential dynamics for the population. The populations that are observed in the models are the AFE population denoted by *n*_*a*_, the NKX2-1^+^ LP population represented by *n*_*p*_, and the total live cells population defined as *n*_*q*_ = *n*_*a*_ + *n*_*p*_. The population of NKX2-1^+^ LPs is indicated with the expression of NKX2-1 GFP marker^1^. The uncertainty of the biological system characterizes this stage of the directed differentiation protocol (Fig. 1A), which is exacerbated by data sparsity due to the high cost of running experiments with daily measurements. Hence, the mathematical models cannot be high-dimensional with respect to the number of their population states.

The model also incorporates the per capita death, *d*_*j*_, and proliferation rates, *b*_*j*_, for each population, *n*_*j*_. It also includes the per capita differentiation rate ${p}_{j{j}^{{\prime} }}$ between one population *n*_*j*_ and another ${n}_{{j}^{{\prime} }}$. Two cellular processes, proliferation and differentiation, are affected by biochemical concentrations, specifically glucose, *c*_*g*_, and lactate *c*_*l*_, in addition to cell populations. The rates are defined as,

5 $$\begin{array}{rcl}{b}_{j}({\bf{N}}(t),{\bf{C}}(t)) & = & {b}_{j}({n}_{j},{c}_{g},{c}_{l}),\\ {p}_{j{j}^{{\prime} }}({\bf{N}}(t),{\bf{C}}(t)) & = & {p}_{j{j}^{{\prime} }}({n}_{j},{c}_{g},{c}_{l}),\\ {d}_{j}({\bf{N}}(t),{\bf{C}}(t)) & = & {\delta }_{j}.\end{array}$$

Here, all the populations and the concentrations are contained in two vectors, **N**(*t*) and **C**(*t*), respectively. As seen in the equation above, we defined the per capita death rates as constant to make the proposed mathematical models structurally identifiable.

Lineage model M0 (Fig. 1B) is defined as,

6 $$\begin{array}{rcl}\frac{{\rm{d}}{n}_{q}}{{\rm{d}}t}(t) & = & {b}_{q}({n}_{q},{c}_{g},{c}_{l}){n}_{q}(t)-{\delta }_{q}{n}_{q}(t)\\ \frac{{\rm{d}}{c}_{g}}{{\rm{d}}t}(t) & = & -{V}_{g}\cdot ({n}_{q})\frac{{c}_{g}}{{c}_{g}+{\bar{c}}_{g}}\\ \frac{{\rm{d}}{c}_{l}}{{\rm{d}}t}(t) & = & {V}_{l}\cdot ({n}_{q})\frac{{c}_{l}}{{c}_{l}+{\bar{c}}_{l}}\end{array}$$

This model has one population, the total population, and two concentrations, glucose and lactate concentrations. Michaelis-Menten kinetics (MMK) govern glucose and lactate dynamics, with *V*_*g*_ and ${\overline{c}}_{g}$ (*V*_*l*_ and ${\overline{c}}_{l}$) being the limiting rates and the half-saturating constants for glucose (lactate)^55^. MMK models enzyme-limited systems, which is consistent with cellular metabolism stages such as glucose uptake^56^, hexokinase reaction^57^, and cytochrome-c oxidase activity^58^, and is widely used in cell population dynamics models^59–61^. The average proliferation rate is defined as,

7 $${b}_{q}({n}_{q},{c}_{g},{c}_{l})={\beta }_{q}\,f({n}_{q},{c}_{g},{c}_{l})$$

Since only the total count is used in this analysis, a differentiation term is not applicable here, as it would not affect the total population.

The more detailed model, lineage model M1, has two different populations, AFE and LPs. They are shown as,

8 $$\begin{array}{rcl}\frac{{\rm{d}}{n}_{a}}{{\rm{d}}t}(t) & = & {b}_{a}({n}_{a},{c}_{g},{c}_{l}){n}_{a}(t)-{\delta }_{a}{n}_{a}(t)-{p}_{a}({n}_{a},{c}_{g},{c}_{l}){n}_{a}(t),\\ \frac{{\rm{d}}{n}_{p}}{{\rm{d}}t}(t) & = & {b}_{p}({n}_{p},{c}_{g},{c}_{l}){n}_{p}(t)-{\delta }_{p}{n}_{p}(t)+{p}_{a}({n}_{a},{c}_{g},{c}_{l}){n}_{a}(t),\\ \frac{{\rm{d}}{c}_{g}}{{\rm{d}}t}(t) & = & -{V}_{g}\cdot ({n}_{a}+{n}_{p})\frac{{c}_{g}}{{c}_{g}+{\bar{c}}_{g}},\\ \frac{{\rm{d}}{c}_{l}}{{\rm{d}}t}(t) & = & {V}_{l}\cdot ({n}_{a}+{n}_{p})\frac{{c}_{l}}{{c}_{l}+{\bar{c}}_{l}}.\end{array}$$

This model assumes the population of other cell types, e.g., definitive endoderm or other by-products, is negligible. Also, it assumes that LPs do not differentiate into later-stage cells; our experimental observations confirm this. Cellular processes are defined here as,

9 $$\begin{array}{rcl}{b}_{a}({n}_{a},{c}_{g},{c}_{l}) & = & {\beta }_{a}f({n}_{a},{c}_{g},{c}_{l}),{p}_{ap}({n}_{a},{c}_{g},{c}_{l})=2(1-{p}_{ap}){b}_{a}({n}_{a},{c}_{g},{c}_{l}),\\ {b}_{p}({n}_{p},{c}_{g},{c}_{l}) & = & {\beta }_{p}f({n}_{p},{c}_{g},{c}_{l}).\end{array}$$

As seen above, *β*_*a*_ and *β*_*p*_ are the maximum proliferation rates for AFE and LPs. Also, AFE proliferates with the rate of *β*_*a*_*f*(*n*_*a*_, *c*_*g*_, *c*_*l*_), that would result in two daughter stem cells with the probability of ${p}_{ap}$ or two differentiated cells with the probability of $(1-{p}_{ap})$ assuming only symmetric division^62,63^. Note that the lineage models are designed so that their states, *n*_*a*_, *n*_*p*_, *n*_*q*_, *c*_*g*_, and *c*_*l*_ are measured in the experiments (Supplementary Table 1).

The one-state formulation, M0, is most appropriate when experimental observations are limited to aggregate live cell densities and extracellular metabolites. In such contexts, the primary objective is rapid forecasting of overall biomass or nutrient demand, and the parsimony of M0 facilitates structural and practical identifiability with sparse data^59,60^. On the other hand, the two-state formulation, M1, resolves the AFE and NKX2-1^+^ LP populations, and therefore requires lineage-specific readouts using flow cytometry facilitated by immunofluorescence staining or reporter lines. When these measurements are available, M1 affords mechanistic insight into the AFE to LP transition and enables optimization of differentiation efficiency^62,64^.

Per capita growth models are modulated by the respective cell population and the biochemical concentrations of glucose and lactate. The total per capita growth is defined as,

10 $$f({n}_{j},{c}_{g},{c}_{l})={F}_{{\mathrm{env}}}({c}_{g},{c}_{l})\cdot {f}_{n}({n}_{j}),$$

where *f*_*n*_(*n*_*j*_) is the per capita growth rate. There are multiple ways to define it,

11 $$\begin{array}{cc}{f}_{n}({n}_{j})=1 & {\mathrm{Exponential}},\\ {f}_{n}({n}_{j})=\left(1-\frac{{n}_{j}}{{n}_{\max }}\right) & {\mathrm{Logistic}},\\ {f}_{n}({n}_{j})=\log \left(\frac{{n}_{\max }}{{n}_{j}}\right) & {\mathrm{Gompertz}}.\end{array}$$

In these equations, ${n}_{\max }$ is the maximum population caused by limited space. Exponential growth means there is no space limit on the growth, and logistic growth means that space causes a linear decrease in the per capita growth rate^21^.

The effect of the biochemical environment, *F*_env_(*c*_*g*_, *c*_*l*_), is defined as a multiplicative effect of each chemical substrate as below^61^.

12 $$\begin{array}{rcl}{F}_{{\mathrm{env}}}({c}_{g},{c}_{l}) & = & {f}_{1}({c}_{g}(t))\cdot {f}_{2}({c}_{l}(t))\\ {\mathrm{GluLac}}:\,\,{F}_{{\mathrm{env}}} & = & \frac{{c}_{g}(t)}{{K}_{g}+{c}_{g}(t)}\cdot \frac{{K}_{l}(t)}{{K}_{l}+{c}_{l}(t)}\\ {\mathrm{Lac}}:\,\,{F}_{{\mathrm{env}}} & = & 1\cdot \frac{{K}_{l}}{{K}_{l}+{c}_{l}(t)}\\ {\mathrm{Glu}}:\,\,{F}_{{\mathrm{env}}} & = & \frac{{c}_{g}(t)}{{K}_{g}+{c}_{g}(t)}\cdot 1\\ {\mathrm{NoEffect}}:\,\,{F}_{{\mathrm{env}}} & = & 1\cdot 1\end{array}$$

Here, two models are assumed for the glucose effect, no effect or positive MMK, and two for the lactate effect, no effect or negative MMK^60,65^. This results in a total of 12 growth models, formed by all combinations of three population-controlled growth models (Eq. (11)) and four environmental effect models (Eq. (12)).

### Error models

Two error functions, one for densities, ${\epsilon }_{{n}_{j}}$, and one for concentrations, ${\epsilon }_{{c}_{j}}$, are defined because these states possess different units. Also, three error model candidates are defined as follows,

13 $$\begin{array}{lll}{\epsilon }_{{n}_{j}}={a}_{n}, & {\epsilon }_{{c}_{j}}={a}_{c}, & {\mathrm{Additive}},\\ {\epsilon }_{{n}_{j}}={b}_{n}\cdot {g}_{j}({\bf{Y}},{\mathbf{\Theta }},{\bf{u}}), & {\epsilon }_{{c}_{j}}={b}_{c}\cdot {g}_{j}({\bf{Y}},{\mathbf{\Theta }},{\bf{u}}), & {\mathrm{Proportional}},\\ {\epsilon }_{{n}_{j}}={a}_{n}+{b}_{n}\cdot {g}_{j}({\bf{Y}},{\mathbf{\Theta }},{\bf{u}}), & {\epsilon }_{{c}_{j}}={a}_{c}+{b}_{c}\cdot {g}_{j}({\bf{Y}},{\mathbf{\Theta }},{\bf{u}}), & {\mathrm{Combined}}.\end{array}$$

Here, *b*_*n*_ and *b*_*c*_ are unitless, while *a*_*n*_ and *a*_*c*_ have the units of density and concentration, respectively.

Note that this study requires error models since data standard deviations are unreliable due to the small number of replicates^18^. As shown by Eq. (13), the error models quantify the relationship between the standard deviations, ***ϵ***, and the expected values, **g**. Individual error models are also justified since the density measurements include human-related errors, while the concentration measurements are affected by instrument errors but entirely independent of human errors. Introducing error models in the model definition necessitates the definition of a general loss function based on the likelihood function to infer the structural and error parameters simultaneously.

### Objective function definition

Parameter estimation using experimental data is done by maximizing the likelihood function, ${\mathcal{L}}$. Model parameters consist of structural parameters and error parameters and are mathematically defined as ***Ψ*** = [**Θ**, ***ξ***]. When dealing with independent observations, *z*_*i*_, the likelihood function is simplified to the multiplication of probability density functions, *p*, as,

14 $${\mathcal{L}}({\mathbf{\Theta }},{\boldsymbol{\xi }};{\bf{z}})=\mathop{\prod }\limits_{i=1}^{n}p({\mathbf{\Theta }},{\boldsymbol{\xi }};{z}_{i}).$$

It should be noted that under our assumption of independent and identically distributed normalized residuals, ${\boldsymbol{\eta }} \sim {\mathcal{N}}({\bf{0}},{\bf{I}})$. Eq. (14) further simplifies to obtaining the inferred structural and error parameters, Θ^*^ and ξ^*^, by minimizing the negative log-likelihood, − *ℓ*, as,

15 $$\begin{array}{rcl}({{\mathbf{\Theta }}}^{* },{{\boldsymbol{\xi }}}^{* }) & = & \arg \mathop{\min }\limits_{{\mathbf{\Theta }},{\boldsymbol{\xi }}}-2\ell ({\mathbf{\Theta }},{\boldsymbol{\xi }};{{\mathcal{S}}}_{T})\\ & = & \arg \mathop{\min }\limits_{{\mathbf{\Theta }},{\boldsymbol{\xi }}}\mathop{\sum }\limits_{{g}_{j}\in {\bf{g}}}\mathop{\sum }\limits_{{Z}_{{j}_{k}}\in {{\mathcal{S}}}_{T}}{\epsilon }_{j}^{-2}({\mathbf{\Theta }},{\boldsymbol{\xi }},{t}_{k}){({Z}_{{j}_{k}}-{g}_{j}({\mathbf{\Theta }},{t}_{k}))}^{2}\\ & & +\mathop{\sum }\limits_{{g}_{j}\in {\bf{g}}}\mathop{\sum }\limits_{{Z}_{{j}_{k}}\in {{\mathcal{S}}}_{T}}2\mathrm{ln}({\epsilon }_{j}({\mathbf{\Theta }},{\boldsymbol{\xi }},{t}_{k}))+\mathop{\sum }\limits_{{g}_{j}\in {\bf{g}}}\mathop{\sum }\limits_{{Z}_{{j}_{k}}\in {{\mathcal{S}}}_{T}}\mathrm{ln}(2\pi ).\end{array}$$

Here, *g*_*j*_ is the model prediction, *Z*_*j*_ is the experimental measurement, $\mathrm{ln}$ is natural logarithm, and ${{\mathcal{S}}}_{T}$ is the training dataset^10,16^. In the case of M0, the total population and the two concentrations are employed in Eq. (15), while for M1, each of the two distinctive populations and the concentrations are used.

### Structural identifiability analysis

A mathematical model is structurally identifiable if it has a unique set of parameters that corresponds to a set of unlimited noiseless observations^19–21^. Mathematically, if two valid sets of structural model parameters are **Θ** and **Φ**, then,

16 $${\bf{g}}({\bf{Y}},{\mathbf{\Theta }},{\bf{u}})={\bf{g}}({\bf{Y}},{\mathbf{\Phi }},{\bf{u}})\,\Rightarrow \,{\mathbf{\Theta }}={\mathbf {\Phi }}.$$

Structural identifiability analysis focuses on the relationship between the model equations and the measured states and is independent of the experimental data^16^. The analysis entails differential algebra techniques to derive input-output equations from the model. This leads to constructing a symbolic identifiability matrix, and if the matrix is of full rank, it would indicate the structural identifiability of the model^22,66^. In the model development procedure, structurally unidentifiable models are discarded or modified^10,67^.

### Model-based design of experimental protocols

We used model-based design of experimental protocols (MBDEP) to determine the sampling period needed to infer an accurate model, as indicated by the error measure, which represents the relative distance between the inferred and assumed parameters. Using Eq. (4) and by choosing specific structural and error models with their corresponding parameters (assumed parameters, $[\widehat{{\mathbf{\Theta }}},\widehat{{\boldsymbol{\xi }}}]$), synthetic data can be generated. By utilizing the generated noisy synthetic data to solve Eq. (15), the inferred structural parameters (Θ^*^) may be obtained. We define *e* as the relative distance measure between the assumed ($\widehat{{\mathbf{\Theta }}}$) and inferred parameters, i.e., parameters utilized to produce synthetic data using the equation below,

17 $$e=\frac{| \widehat{{\mathbf{\Theta }}}-{{\mathbf{\Theta }}}^{* }| }{| \widehat{{\mathbf{\Theta }}}| }.$$

The procedure was repeated to perform a grid search at various sampling intervals, with the sampling interval that minimizes the error, *e*, chosen for the experiments.

### Model inference

A version of adaptive differential evolution (ADE), formally known as DE/rand/1/bin with radius-limited sampling^68,69^, was used for each optimization run (BlackBoxOptim.jl^70^). ADE methods have demonstrated exceptional capabilities in handling nonlinear, multimodal, and constrained global optimization problems^71,72^. Since ADE does not guarantee local convergence with a given error bound, the solution from ADE is taken as an initial guess for a local optimizer (Nelder-Mead^73,74^) until convergence to the specified tolerance, 10^−6^, is achieved. Note that the choice of Nelder-Mead was made after testing multiple alternatives, including gradient-based methods such as BFGS^75^ and Adam^76^ in this specific use case.

The selection dataset, ${{\mathcal{S}}}_{S}$, is used to calculate the Bayesian information criterion (BIC), defined as,

18 $${\mathrm{BIC}}=k{\mathrm{ln}}({m}_{s})-2\ell ({\mathbf{\Theta }},{\boldsymbol{\xi }};{{\mathcal{S}}}_{S}),$$

where *k* and *m*_*s*_ are the number of inferred parameters and the number of observations in the selection dataset. This measure is calculated on all candidate models on the inferred parameter values, and minimizing it strikes a balance between model complexity and prediction accuracy^77^.

### Practical identifiability analysis

Practical identifiability extends structural identifiability analysis by incorporating experimental data into the assessment of the uniqueness of the inferred parameters. Here, one model parameter, *ψ*_*i*_, is fixed at a value, and the likelihood is maximized on the rest of the parameters (*ψ*_*j*_, ∀ *j* ≠ *i*) in Eq. (15), i.e.,

19 $${\mathrm{PL}}\,({\psi }_{i})=\mathop{\max }\limits_{{\psi }_{j\ne i}}\ell ({\mathbf{\Psi }}).$$

Likelihood profile is generated by repeating this for different values of the fixed parameter *ψ*_*i*_, leading to the confidence interval for each parameter,

20 $${\mathrm{CI}}_{{\mathrm{PL}}}({\psi }_{i})=\{{\psi }_{i}| {\mathrm{PL}}({\psi }_{i})\ge {\ell }_{\max }-{\Delta }_{\alpha }\},$$

where ${\ell }_{\max }$ and Δ_*α*_ are the maximum log-likelihood and *α*-quantile of the *χ*^2^ distribution with one degree of freedom, respectively^16^. Finite confidence intervals signal practically identifiable parameters^32,78^.

### Goodness of fit

The inferred model is validated on the validation (hold-out) dataset, ${{\mathcal{S}}}_{V}$, using the root-mean-squared error (RMSE).

21 $${\mathrm{RMSE}}=\sqrt{\frac{1}{m}\mathop{\sum }\limits_{{g}_{j}\in {\bf{g}}}\mathop{\sum }\limits_{{Z}_{{j}_{k}}\in {{\mathcal{S}}}_{V}}{({Z}_{{j}_{k}}-{g}_{j}({\mathbf{\Theta }},{t}_{k}))}^{2}}.,$$

In this equation, *n*_*z*_, *n*_*k*_, and *m* are the number of data points in each state, the number of states, and the total number of data points, respectively^79^. Further, Normalized RMSE can be defined as,

22 $${\mathrm{NRMSE}}=\frac{1}{\max ({\bf{Z}})-\min ({\bf{Z}})}{\mathrm{RMSE}}$$

where **Z**, shows the experimental data vector^80^.

### Global sensitivity analysis

In this work, global sensitivity analysis (GSA) is employed to rank model parameters based on their impact on predictions. Variance-based Sobol’s method is a GSA method that decomposes nonlinear continuous functions into a set of integrals as,

23 $${Y}_{k}={f}_{0}+\mathop{\sum }\limits_{i=1}^{d}{f}_{i}({X}_{i})+\mathop{\sum }\limits_{i < j}^{d}{f}_{ij}({X}_{i},{X}_{j})+\ldots +{f}_{1,2,\ldots ,d}({X}_{1},{X}_{2},\ldots ,{X}_{d})$$

These integrals increase in dimensionality, representing the overall mean, *f*_0_, main effects, ${f}_{i}({X}_{i})$, and interactions of increasing order between variables^35,81^. Here, ${f}_{ij}({X}_{i},{X}_{j})$ is the first-order interaction between *X*_*i*_ and *X*_*j*_. Note that *d* is the size of **X**, which is a vector that includes the inputs under study, i.e., model parameters, initial conditions, or boundary conditions.

The bounds for sensitivity analysis were calculated using $\exp (\log ({\theta }^{* })\pm \log (l))=[{\theta }^{* }/l,{\theta }^{* }\cdot l]$ to have θ^*^ at the center of the log-scaled search space. Note that θ^*^ stands for the inferred parameter values. The span for the bounds was chosen as *l* = 1.1. For a more in-depth look into the methodology (Fig. 1C), refer to previous works by the authors^16,17^.

## Supplementary information

Supplementary Information

## Data Availability

All data and code used to generate the results in this manuscript are available through https://github.com/amostof/inSilicoAFEPaper.

## Code availability

All data and code used to generate the results in this manuscript are available through https://github.com/amostof/inSilicoAFEPaper.
