# Supplementary material for: In silico modeling of anterior foregut endoderm differentiation towards lung epithelial progenitors
Source: NPJ Syst Biol Appl. 2026 Jan 26;12:29. doi: 10.1038/s41540-026-00650-1 (PMC12920931; doi:10.1038/s41540-026-00650-1)
Supplement: Supplementary file 1 — Supplementary Information [file 41540_2026_650_MOESM1_ESM.pdf]

Supplementary Information

In silico modeling of anterior foregut endoderm differentiation towards lung epithelial progenitors

Amirmahdi Mostofinejad[1], David A. Romero[1], Dana Brinson[2,3], Thomas K. Waddell[2,3,4], Golnaz Karoubi[1,2,5], and Cristina H. Amon*[1,2]

[1]Department of Mechanical and Industrial Engineering, University of Toronto, Toronto, ON, Canada
[2]Institute of Biomedical Engineering, University of Toronto, Toronto, ON, Canada
[3]Latner Thoracic Surgery Research Laboratories, Toronto General Hospital Research Institute, University Health Network, Toronto, ON, Canada
[4]Institute of Medical Sciences, University of Toronto, Toronto, ON, Canada
[5]Department of Laboratory Medicine and Pathobiology, University of Toronto, Toronto, ON, Canada

Supplementary Table 1: Measurements. The columns show the observables and their corresponding models, and the rows show the measurement time.

Time (day)	Live (M0)	AFE (M1)	LP (M1)	Glucose (Both)	Lactate (Both)
0	✓	✓	✓	✓	✓
1				✓	✓
2	✓	✓	✓	✓	✓
3				✓	✓
4	✓	✓	✓	✓	✓

*Corresponding author

Supplementary Table 2: Model parameters for model-based experimental design of AFE differentiation.

Parameter	Value (p_0)	Unit
b_n	0.3	dimensionless
b_c	0.3	dimensionless
β_s	1	d^{-1}
p_{sd}	0.5	dimensionless
δ_s	1	d^{-1}
β_d	1	d^{-1}
δ_d	1	d^{-1}
$n_{\max}$	200	cell mm^{-2}
V_g	1	$\mathrm{mol\,mm}^{-3}$
V_l	1	$\mathrm{mol\,mm}^{-3}$
$\bar{c}_g$	1	$\mathrm{mol\,mm}^{-3}$
$\bar{c}_l$	1	$\mathrm{mol\,mm}^{-3}$
K_g	1	$\mathrm{mol\,mm}^{-3}$
K_l	1	$\mathrm{mol\,mm}^{-3}$

Supplementary Table 3: Value and confidence intervals (lower bound, higher bound) for inferred M1 models. NF stands for not found in the search space, which is interpreted as unidentifiable.

Parameter	Exponential Glu w/ additive	Gompertz w/ proportional	Logistic w/ additive	Logistic w/ proportional
β_a	10.581<br>(10.392, 10.728)	3.154<br>(2.678, 15.86)	6.084<br>(4.409, NF)	12.98<br>(6.931, 47.61)
p_{ap}	0.79749<br>(0.79185, 0.80181)	0.5611<br>(0.5103, NF)	0.5889<br>(0.5060, 0.7123)	0.8450<br>(0.6666, NF)
δ_a	2.0019<br>(1.9709, 2.0422)	1.040<br>(0.5020, 2.233)	0.6811<br>(0.4565, 9.317)	8.238<br>(5.828, 10.39)
β_p	1.1782<br>(-2.9756, 3.5809)	0.7070<br>(-2.020, 18.74)	0.4432<br>(-9.445, 15.80)	1.492<br>(-2.870, 43.87)
δ_p	3.8694<br>(3.1214, 5.3502)	24.07<br>(18.39, 266.0)	9.550<br>(5.970, 56.39)	11.17<br>(7.464, NF)
V_g	0.12569<br>(0.10817, 0.14036)			
K_g	29.563<br>(28.946, 30.392)			
$\bar{c}_g$	398.52<br>(355.59, 465.84)			
$n_{\max}$		5592<br>(3579, NF)	1314<br>(785.8, 57240)	4298<br>(3261, NF)
a_n	89.768<br>(76.696, 106.43)		96.56<br>(82.70, 115.2)	
a_c	0.99004<br>(0.84715, 1.1998)			
b_n		0.7602<br>(0.6292, 1.010)		0.7366<br>(0.5916, 0.9773)

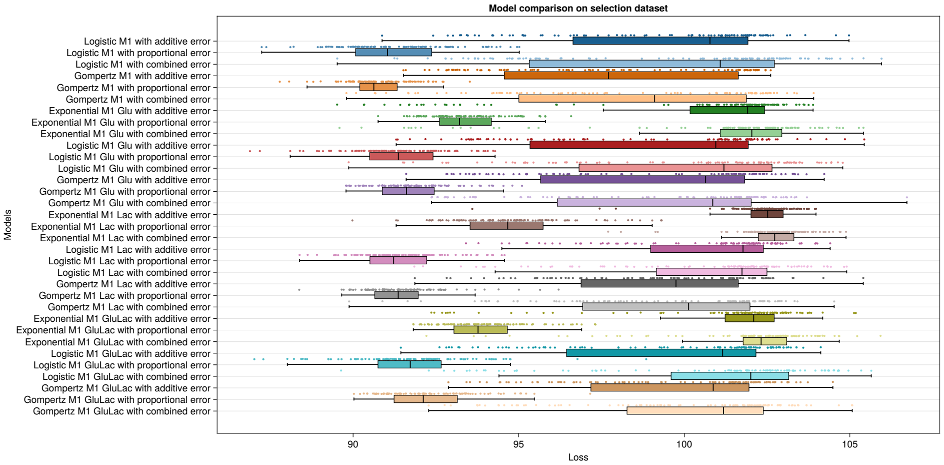

Supplementary Figure 1: **M1 model comparison based on selection loss.** Note that since the number of parameters now does not negatively affect the error measure, the larger models (lower rows) are preferred.

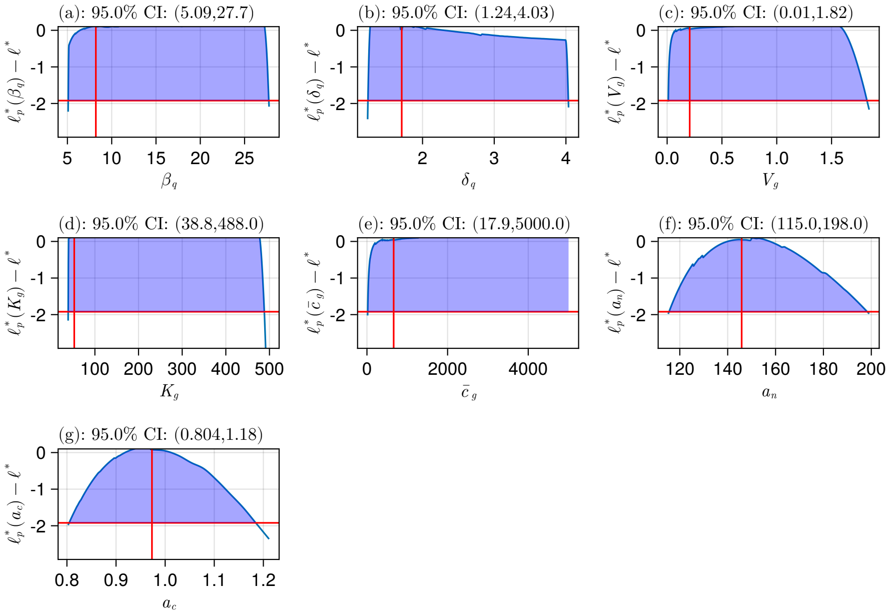

Supplementary Figure 2: **Likelihood profile for exponential M0 Glu model with additive error.**

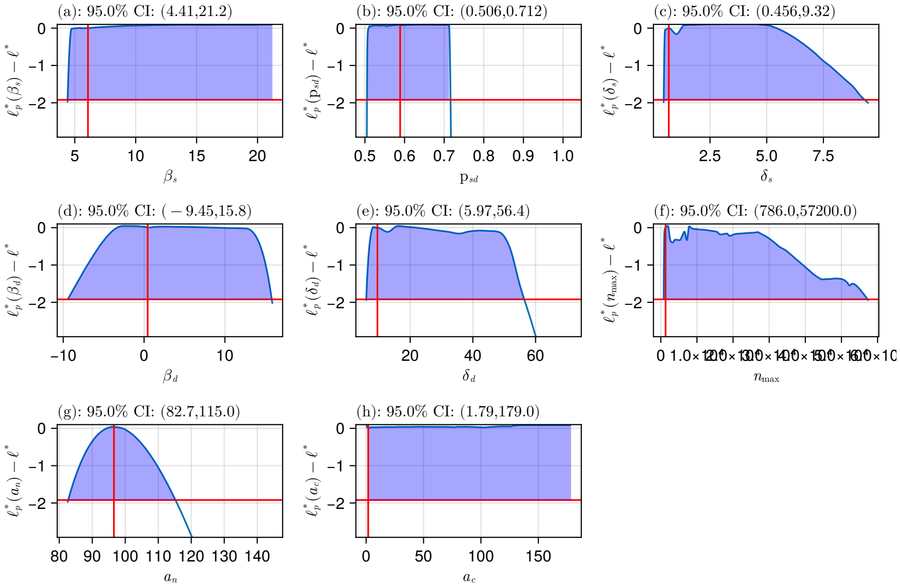

Supplementary Figure 3: **Likelihood profile for logistic M1 model with additive error.**

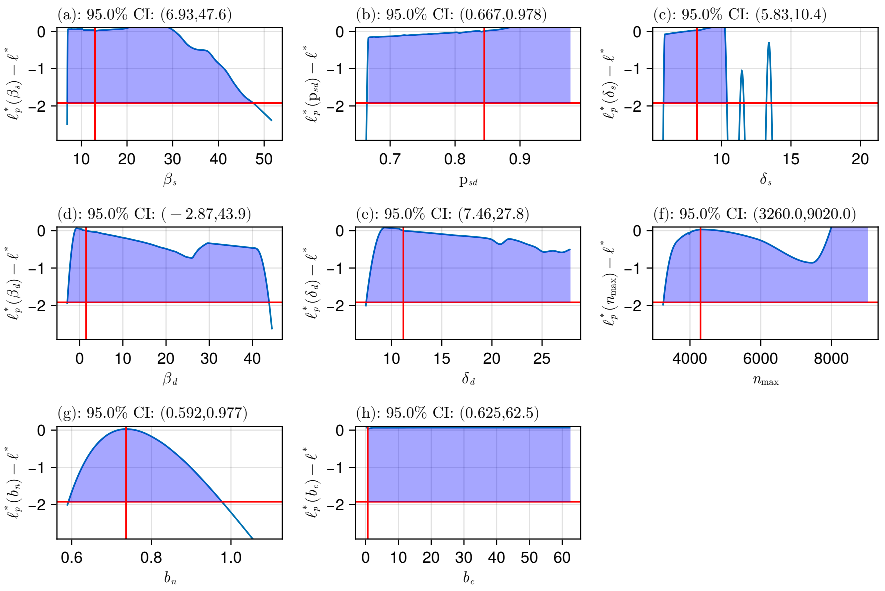

Supplementary Figure 4: **Likelihood profile for logistic M1 model with proportional error.**

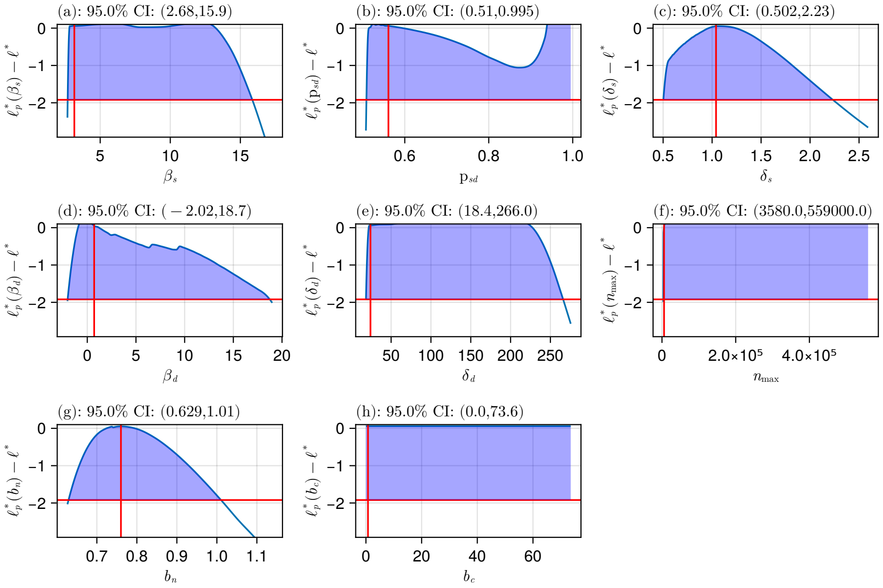

Supplementary Figure 5: **Likelihood profile for Gompertz M1 model with proportional error.**

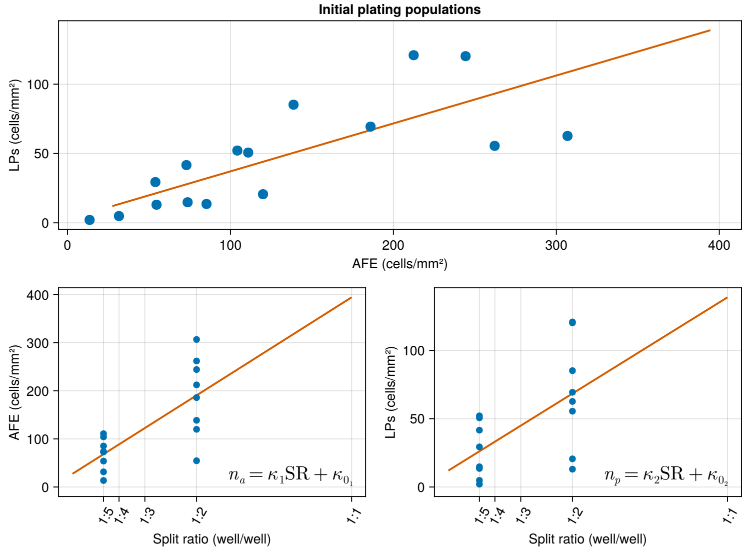

Supplementary Figure 6: **Mapping day 10 split ratio to day 11 populations of the directed differentiation protocol.** Orange lines show the inferred mappings, and the blue dots show the experimental data used for the mapping. The bottom plots show each mapping, while the top figure depicts the resulting correlation between day 11 AFE and LP populations.

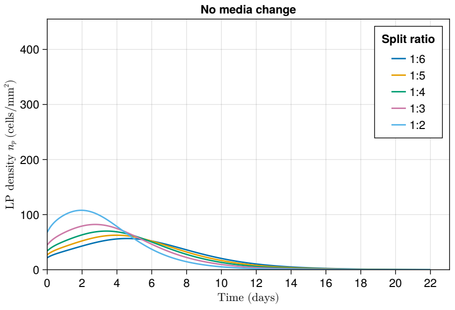

(a) No media change (MCH0).

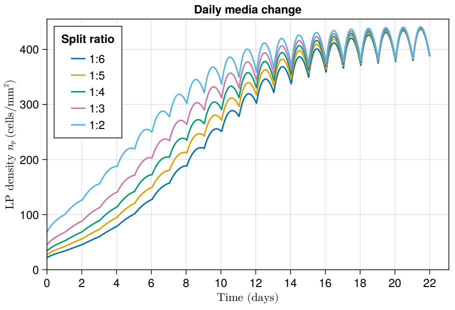

(b) Daily media change (MCH1).

Supplementary Figure 7: **Time evolution of LP density in different split ratios and media change conditions.**
